# The genome sequence of Trimmer's Mining Bee, *Andrena trimmerana *(Kirby, 1802)

**DOI:** 10.12688/wellcomeopenres.23370.1

**Published:** 2024-11-22

**Authors:** Ellen Baker, Liam M. Crowley, Steven Falk, University of Oxford and Wytham Woods Genome Acquisition Lab, Darwin Tree of Life Barcoding collective, Wellcome Sanger Institute Tree of Life Management, Samples and Laboratory team, Wellcome Sanger Institute Scientific Operations: Sequencing Operations, Wellcome Sanger Institute Tree of Life Core Informatics team, Tree of Life Core Informatics collective, Darwin Tree of Life Consortium

**Affiliations:** 1 University of Oxford, Oxford, England, UK; 2 Independent researcher, Kenilworth, England, UK

**Keywords:** Andrena trimmerana, Trimmer's Mining Bee, genome sequence, chromosomal, Hymenoptera

## Abstract

We present a genome assembly from afemale specimen of *Andrena trimmerana* (Trimmer’s Mining Bee; Arthropoda; Insecta; Hymenoptera; Andrenidae). The genome sequence has a total length of 399.10 megabases. Most of the assembly (86.18%) is scaffolded into 5 chromosomal pseudomolecules. The mitochondrial genome has also been assembled and is 19.77 kilobases in length. Gene annotation of this assembly on Ensembl identified 10,570 protein-coding genes.

## Species taxonomy

Eukaryota; Opisthokonta; Metazoa; Eumetazoa; Bilateria; Protostomia; Ecdysozoa; Panarthropoda; Arthropoda; Mandibulata; Pancrustacea; Hexapoda; Insecta; Dicondylia; Pterygota; Neoptera; Endopterygota; Hymenoptera; Apocrita; Aculeata; Apoidea; Anthophila; Andrenidae; Andreninae; *Andrena*; *Hoplandrena; Andrena trimmerana* (Kirby, 1802) (NCBI:txid1431430).

## Background

*Andrena trimmerana*, or Trimmer’s Mining Bee, is a solitary bee species within the family Andrenidae. It is widely distributed across western Europe, including Britain and Ireland, and extends into parts of western Asia and North Africa ( Else, 2023; GBIF Secretariat, 2024). In the UK, this species is found from East Kent to Cornwall and northwards to the Midlands, with isolated records in the Lake District. It is generally absent from Scotland ( NBN Atlas Partnership, 2024). However, some recorders aggregate sightings of *A. trimmerana* and *A. scotica* due to the difficulty in distinguishing them ( Else, 2023).

*Andrena trimmerana* inhabits a variety of environments such as coastal cliffs, open woodlands, heathlands, chalk grasslands, fens, commons, and gardens ( Else, 2023). The species prefers well-drained soils and typically nests individually in soil banks and bare slopes, rather than forming large nesting aggregations. The species is polylectic and visits willow, dandelion, gorse, bramble, blackthorn, alexanders, and rhododendron ( Else, 2023).

The genus *Andrena* is taxonomically challenging because of the morphological similarity across species and the variation between broods in bivoltine species ( Wood *et al.*, 2022). *A. trimmerana* typifies this problem. In addition to being difficult to separate from *A. scotica*, it it is bivoltine, with morphologically distinct spring and summer broods, particularly in males. The broods have previously been classified as different species and there is a possibility that they may be ( Else, 2023).

An extensive DNA barcode survey of wild urban bees in France showed that *Andrena trimmerana*, *A. carantonica*, *A. scotica* and *A. spinigera* share a Barcode Index Number (BIN), suggesting the need for taxonomic revision to establish the status of these species ( Villalta *et al.*, 2021). Documenting the genome sequence of *A. trimmerana* within the Darwin Tree of Life project supports accurate species identification and improves understanding of solitary bee ecology, aiding pollinator conservation across the UK and Ireland.

## Genome sequence report

**Figure 1.**
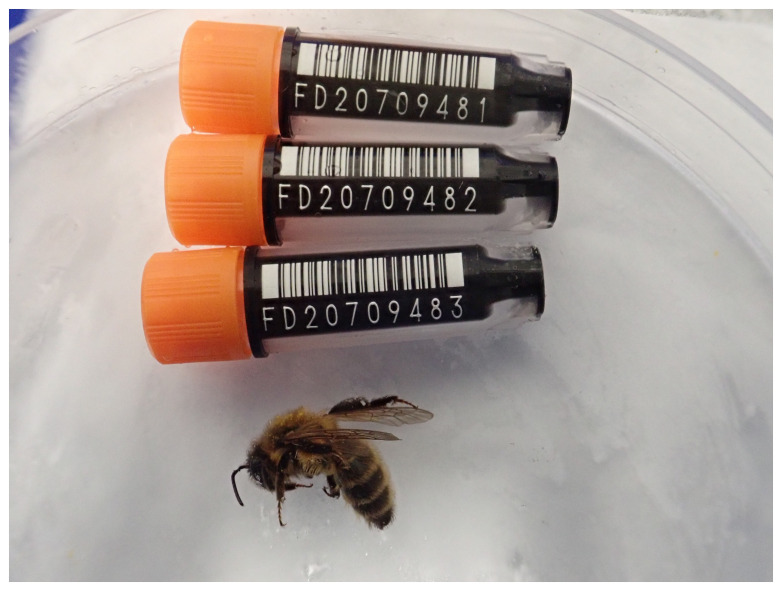
Photograph of the *Andrena trimmerana* (iyAndTrim1) specimen used for genome sequencing.

The genome of *Andrena trimmerana* ( Figure 1) was sequenced using Pacific Biosciences single-molecule HiFi long reads, generating a total of 23.95 Gb (gigabases) from 2.30 million reads, providing an estimated 61-fold coverage. Primary assembly contigs were scaffolded with chromosome conformation Hi-C data, which produced 107.78 Gb from 713.76 million reads. Specimen and sequencing details are summarised in Table 1.

**Table 1.** Specimen and sequencing data for *Andrena trimmerana*.

Project information			
**Study title**	Andrena trimmerana (trimmer's mining bee)		
**Umbrella BioProject**	PRJEB56904		
**Species**	*Andrena trimmerana*		
**BioSample**	SAMEA10107041		
**NCBI taxonomy ID**	1431430		
Specimen information			
**Technology**	**ToLID**	**BioSample ** **accession**	**Organism part**
**PacBio long read sequencing**	iyAndTrim1	SAMEA10200733	thorax
**Hi-C sequencing**	iyAndTrim2	SAMEA10201024	head
**RNA sequencing**	iyAndTrim1	SAMEA10200734	abdomen
Sequencing information			
**Platform**	**Run ** **accession**	**Read count**	**Base count ** **(Gb)**
**Hi-C Illumina NovaSeq 6000**	ERR10395991	7.14e+08	107.78
**PacBio Sequel IIe**	ERR10439742	2.30e+06	23.95
**RNA Illumina NovaSeq 6000**	ERR10395992	7.77e+07	11.74

**Figure 2.**
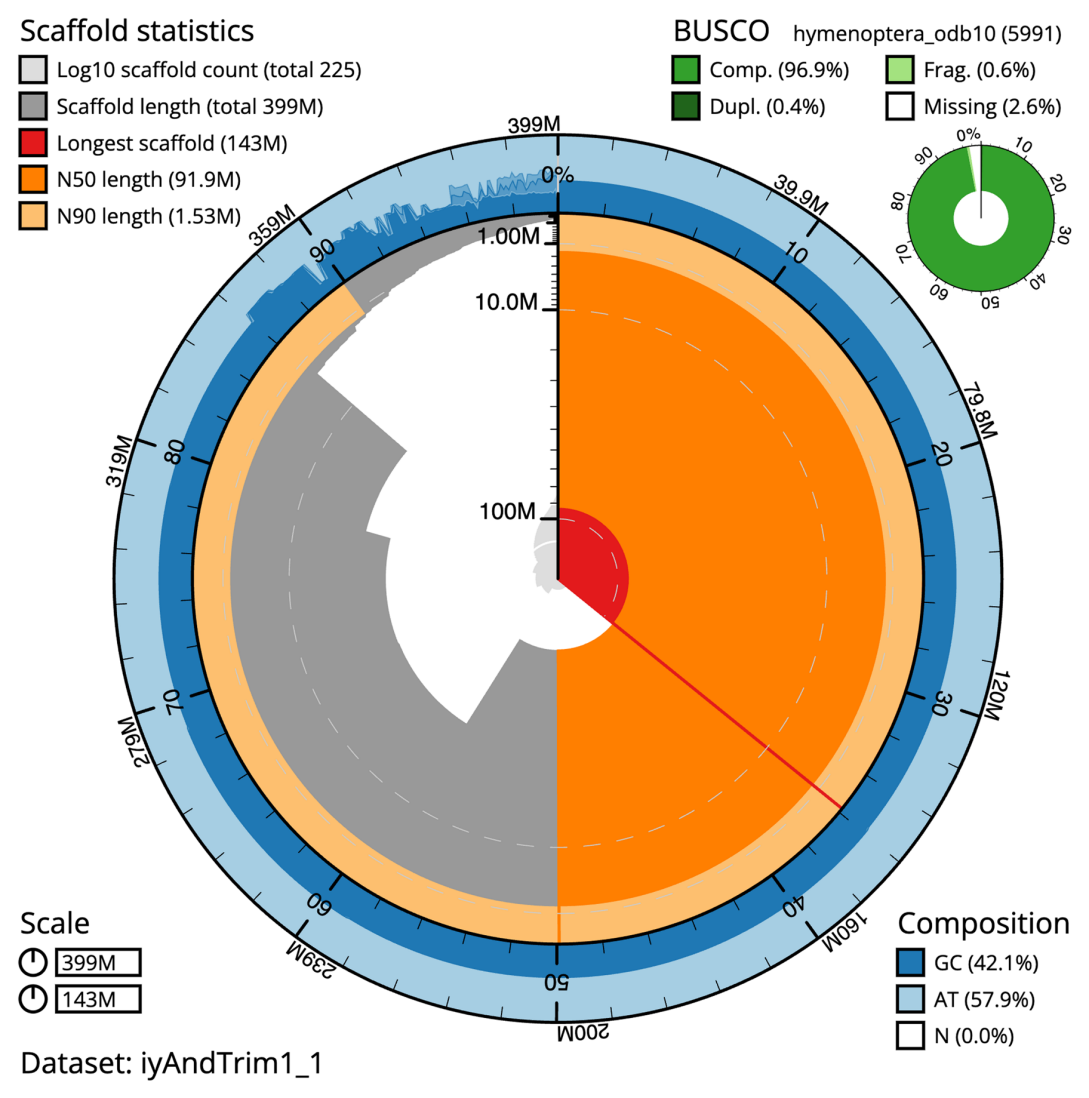
Genome assembly of *Andrena trimmerana*, iyAndTrim1.1: metrics. The BlobToolKit snail plot shows N50 metrics and BUSCO gene completeness. The main plot is divided into 1,000 bins around the circumference with each bin representing 0.1% of the 399,083,795 bp assembly. The distribution of scaffold lengths is shown in dark grey with the plot radius scaled to the longest scaffold present in the assembly (142,895,829 bp, shown in red). Orange and pale-orange arcs show the N50 and N90 scaffold lengths (91,870,870 and 1,525,000 bp), respectively. The pale grey spiral shows the cumulative scaffold count on a log scale with white scale lines showing successive orders of magnitude. The blue and pale-blue area around the outside of the plot shows the distribution of GC, AT and N percentages in the same bins as the inner plot. A summary of complete, fragmented, duplicated and missing BUSCO genes in the hymenoptera_odb10 set is shown in the top right. An interactive version of this figure is available at https://blobtoolkit.genomehubs.org/view/Andrena%20trimmerana/dataset/iyAndTrim1_1/snail.

**Figure 3.**
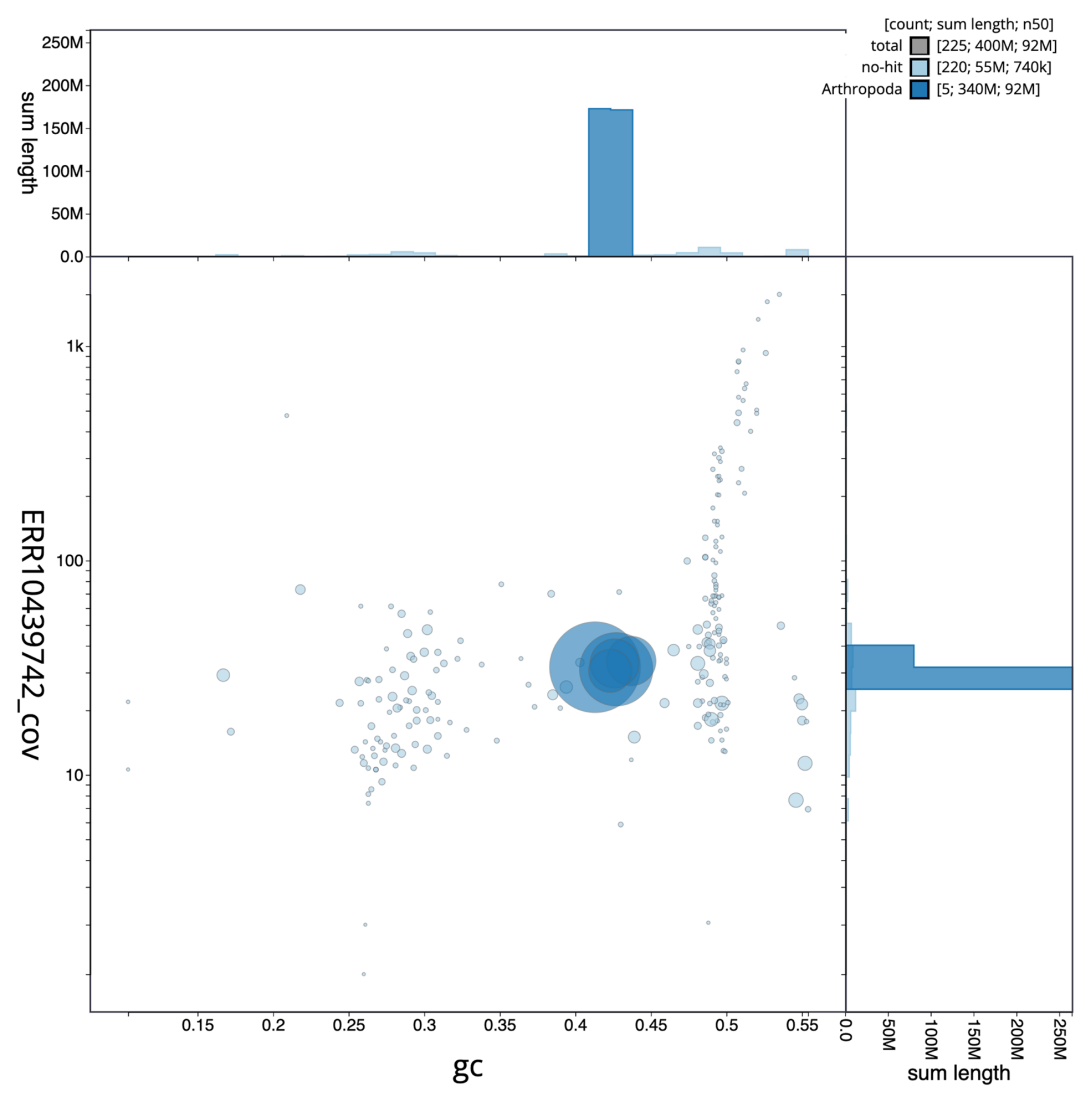
Genome assembly of *Andrena trimmerana*, iyAndTrim1.1: BlobToolKit GC-coverage plot. Sequences are coloured by phylum. Circles are sized in proportion to sequence length. Histograms show the distribution of sequence length sum along each axis. An interactive version of this figure is available at https://blobtoolkit.genomehubs.org/view/Andrena%20trimmerana/dataset/iyAndTrim1_1/blob.

**Figure 4.**
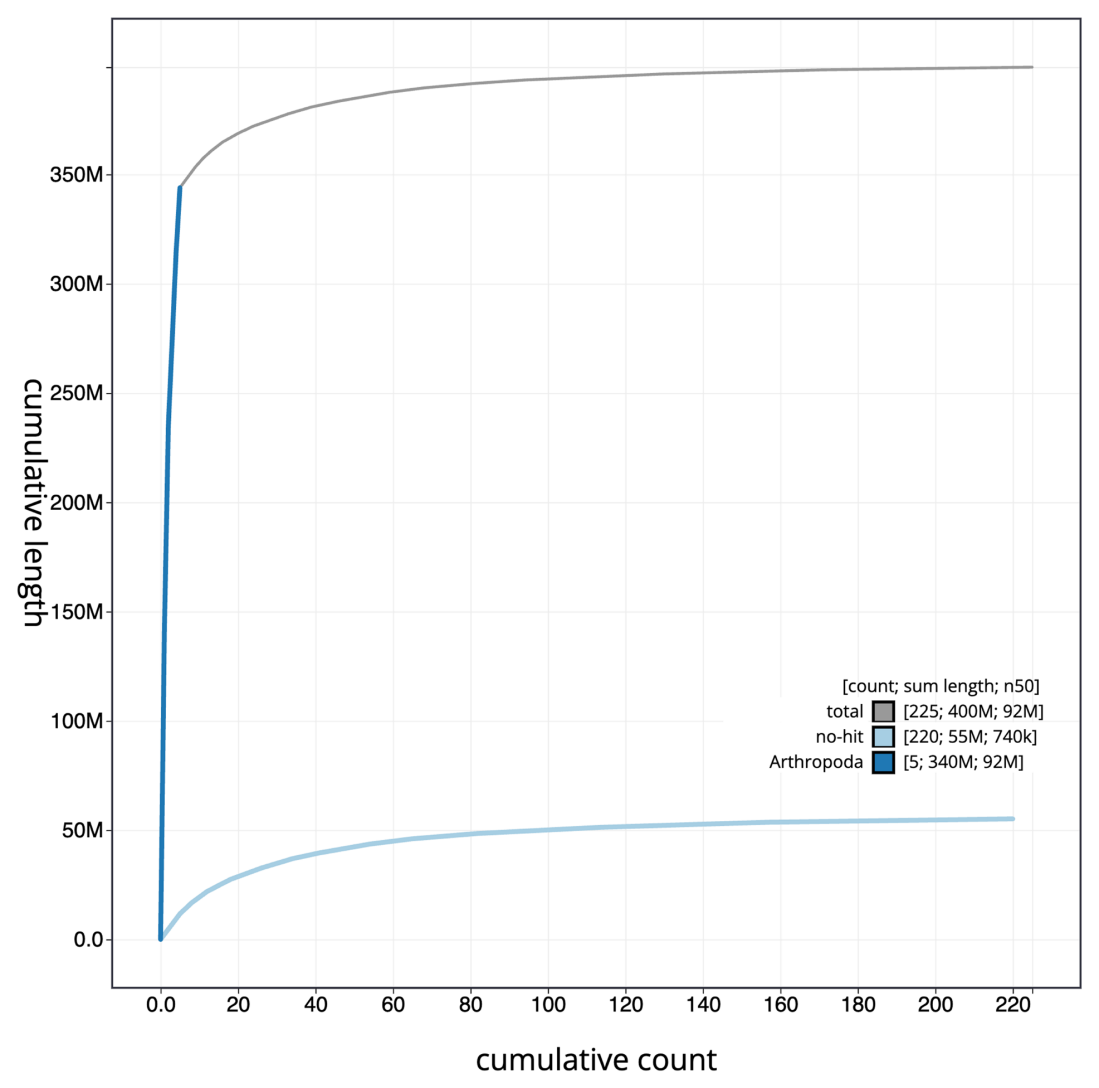
Genome assembly of *Andrena trimmerana* iyAndTrim1.1: BlobToolKit cumulative sequence plot. The grey line shows cumulative length for all sequences. Coloured lines show cumulative lengths of sequences assigned to each phylum using the buscogenes taxrule. An interactive version of this figure is available at https://blobtoolkit.genomehubs.org/view/Andrena%20trimmerana/dataset/iyAndTrim1_1/cumulative.

Assembly errors were corrected by manual curation, including 15 missing joins or mis-joins and haplotypic duplications. This reduced the scaffold number by 5.06%, and increased the scaffold N50 by 48.51%. The final assembly has a total length of 399.10 Mb in 224 sequence scaffolds, with 27 gaps, and a scaffold N50 of 91.9 Mb ( Table 2). The snail plot in Figure 2 provides a summary of the assembly statistics, indicating the distribution of scaffold lengths and other assembly metrics. Figure 3 shows the distribution of scaffolds by GC proportion and coverage. Figure 4 presents a cumulative assembly plot, with separate curves representing different scaffold subsets assigned to various phyla, illustrating the completeness of the assembly.

**Table 2.** Genome assembly data for *Andrena trimmerana*, iyAndTrim1.1.

Genome assembly		
Assembly name	iyAndTrim1.1	
Assembly accession	GCA_951215215.1	
*Accession of alternate haplotype*	*GCA_951212825.1*	
Span (Mb)	399.10	
Number of contigs	252	
Number of scaffolds	224	
Longest scaffold (Mb)	142.9	
Assembly metrics *		*Benchmark*
Contig N50 length (Mb)	17.8	*≥ 1 Mb*
Scaffold N50 length (Mb)	91.9	*= chromosome N50*
Consensus quality (QV)	65.2	*≥ 40*
*k*-mer completeness	100.0%	*≥ 95%*
BUSCO v5.4.3 lineage: hymenoptera_odb10	C:96.9%[S:96.5%,D:0.4%], F:0.6%,M:2.5%,n:5991	*S > 90%*, *D < 5%*
Percentage of assembly mapped to chromosomes	86.18%	*≥ 90%*
Sex chromosomes	None	*localised homologous pairs*
Organelles	Mitochondrial genome: 19.77 kb	*complete single alleles*
Genome annotation of assembly GCA_951215215.1 at Ensembl		
Number of protein-coding genes	10,570	
Number of non-coding genes	1,621	
Number of gene transcripts	18,433	

* Assembly metric benchmarks are adapted from Rhie *et al.* (2021) and the Earth BioGenome Project Report on Assembly Standards September 2024. ** BUSCO scores based on the hymenoptera_odb10 BUSCO set using version 5.3.2. C = complete [S = single copy, D = duplicated], F = fragmented, M = missing, n = number of orthologues in comparison. A full set of BUSCO scores is available at https://blobtoolkit.genomehubs.org/view/Andrena%20trimmerana/dataset/iyAndTrim1_1/busco.

**Figure 5.**
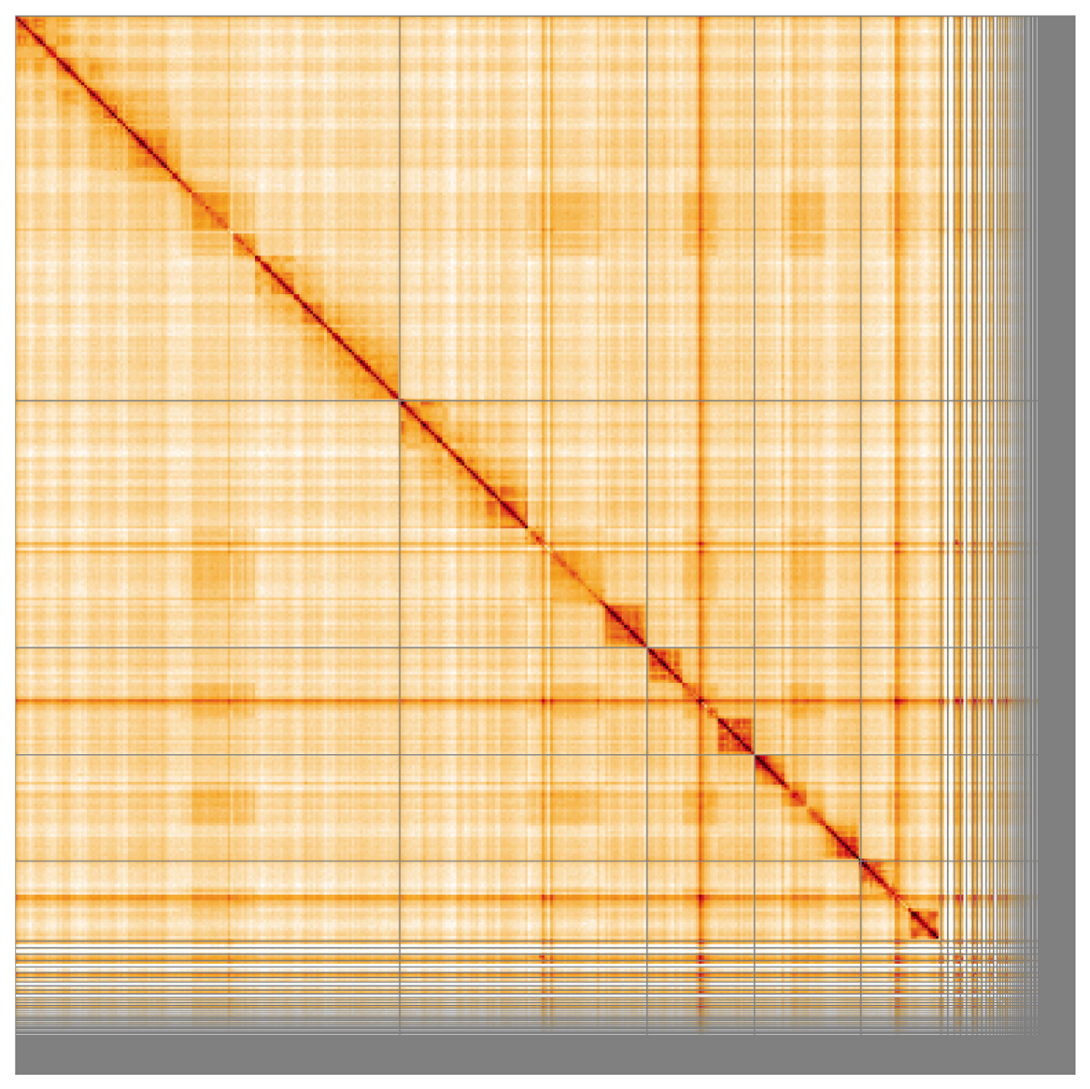
Genome assembly of *Andrena trimmerana*, iyAndTrim1.1: Hi-C contact map of the iyAndTrim1.1 assembly, visualised using HiGlass. Chromosomes are shown in order of size from left to right and top to bottom. An interactive version of this figure may be viewed at https://genome-note-higlass.tol.sanger.ac.uk/l/?d=LwthMvtpQ6aOYoK0J8qqsQ.

Most of the assembly sequence (86.18%) was assigned to 5 chromosomal-level scaffolds. These chromosome-level scaffolds, confirmed by the Hi-C data, are named in order of size ( Figure 5; Table 3). 

**Table 3.** Chromosomal pseudomolecules in the genome assembly of *Andrena trimmerana*, iyAndTrim1.

INSDC accession	Name	Length (Mb)	GC%
OX578059.1	1	142.9	41.5
OX578060.1	2	91.87	42.5
OX578061.1	3	39.86	43.5
OX578062.1	4	39.55	42.5
OX578063.1	5	29.78	42.5
OX578064.1	MT	0.02	21.0

While not fully phased, the assembly deposited is of one haplotype. Contigs corresponding to the second haplotype have also been deposited. The mitochondrial genome was also assembled and can be found as a contig within the multifasta file of the genome submission.

The estimated Quality Value (QV) of the final assembly is 65.2 with *k*-mer completeness of 100.0%, and the assembly has a BUSCO v5.3.2 completeness of 96.9% (single = 96.5%, duplicated = 0.4%), using the hymenoptera_odb10 reference set ( *n* = 5,991). The assembly achieves the EBP reference standard of 6.7.65.2. Other quality metrics are given in Table 2. 

Metadata for specimens, barcode results, spectra estimates, sequencing runs, contaminants and pre-curation assembly statistics are given at https://links.tol.sanger.ac.uk/species/1431430.

## Genome annotation report

The *Andrena trimmerana* genome assembly (GCA_951215215.1) was annotated at the European Bioinformatics Institute (EBI) on Ensembl Rapid Release. The resulting annotation includes 18,433 transcribed mRNAs from 10,570 protein-coding and 1,621 non-coding genes ( Table 2; https://rapid.ensembl.org/Andrena_trimmerana_GCA_951215215.1/Info/Index). The average transcript length is 12,564.09. There are 1.51 coding transcripts per gene and 6.57 exons per transcript.

## Methods

### Sample acquisition and DNA barcoding

A female adult *Andrena trimmerana* (specimen ID Ox001117, ToLID iyAndTrim1) was collected from Wytham Woods, Oxfordshire, UK (latitude 51.78, longitude –1.32) on 2021-04-01 by netting. The specimen was collected by Ellen Baker (University of Oxford), identified by Liam Crowley (University of Oxford) and then preserved on dry ice.

The specimen used for Hi-C sequencing (specimen ID Ox001294, ToLID iyAndTrim2) was an adult specimen, netted in the same location on 2021-04-23. The specimen was collected and identified by Steven Falk (independent researcher) and preserved on dry ice.

The initial identification was verified by an additional DNA barcoding process according to the framework developed by Twyford *et al.* (2024). A small sample was dissected from the specimens and stored in ethanol, while the remaining parts were shipped on dry ice to the Wellcome Sanger Institute (WSI). The tissue was lysed, the COI marker region was amplified by PCR, and amplicons were sequenced and compared to the BOLD database, confirming the species identification ( Crowley *et al.*, 2023). Following whole genome sequence generation, the relevant DNA barcode region was also used alongside the initial barcoding data for sample tracking at the WSI ( Twyford *et al.*, 2024). The standard operating procedures for Darwin Tree of Life barcoding have been deposited on protocols.io ( Beasley *et al.*, 2023).

### Nucleic acid extraction

The workflow for high molecular weight (HMW) DNA extraction at the Wellcome Sanger Institute (WSI) Tree of Life Core Laboratory includes a sequence of procedures: sample preparation and homogenisation, DNA extraction, fragmentation and purification. Detailed protocols are available on protocols.io ( Denton *et al.*, 2023b). In sample preparation, the iyAndTrim1 sample was weighed and dissected on dry ice ( Jay *et al.*, 2023). Tissue from the thorax was homogenised using a PowerMasher II tissue disruptor ( Denton *et al.*, 2023a). HMW DNA was extracted in the WSI Scientific Operations core using the Automated MagAttract v2 protocol ( Oatley *et al.*, 2023). The DNA was sheared into an average fragment size of 12–20 kb in a Megaruptor 3 system ( Bates *et al.*, 2023). Sheared DNA was purified by solid-phase reversible immobilisation, using AMPure PB beads to sample to eliminate shorter fragments and concentrate the DNA ( Strickland *et al.*, 2023). The concentration of the sheared and purified DNA was assessed using a Nanodrop spectrophotometer and Qubit Fluorometer and Qubit dsDNA High Sensitivity Assay kit. Fragment size distribution was evaluated by running the sample on the FemtoPulse system.

RNA was extracted from abdomen tissue of iyAndTrim1 in the Tree of Life Laboratory at the WSI using the RNA Extraction: Automated MagMax™ *mir*Vana protocol ( do Amaral *et al.*, 2023). The RNA concentration was assessed using a Nanodrop spectrophotometer and a Qubit Fluorometer using the Qubit RNA Broad-Range Assay kit. Analysis of the integrity of the RNA was done using the Agilent RNA 6000 Pico Kit and Eukaryotic Total RNA assay.

### Sequencing

Pacific Biosciences HiFi circular consensus DNA sequencing libraries were constructed according to the manufacturers’ instructions. Poly(A) RNA-Seq libraries were constructed using the NEB Ultra II RNA Library Prep kit. DNA and RNA sequencing was performed by the Scientific Operations core at the WSI on Pacific Biosciences Sequel IIe (HiFi) and Illumina NovaSeq 6000 (RNA-Seq) instruments.

Hi-C data were generated from frozen whole organism tissue of iyAndTrim2, using the Arima-HiC v2 kit. The tissue was fixed with a TC buffer containing formaldehyde, resulting in crosslinked DNA. The crosslinked DNA was digested with a restriction enzyme master mix. The resulting 5’-overhangs were filled in and labelled with a biotinylated nucleotide. The biotinylated DNA was then fragmented, enriched, barcoded, and amplified using the NEBNext Ultra II DNA Library Prep Kit. Hi-C sequencing was performed on an Illumina NovaSeq 6000 instrument, using paired-end sequencing with a read length of 150 bp.

### Genome assembly and curation

The HiFi reads were first assembled using Hifiasm ( Cheng *et al.*, 2021) with the --primary option. Haplotypic duplications were identified and removed using purge_dups ( Guan *et al.*, 2020). The Hi-C reads were mapped to the primary contigs using bwa-mem2 ( Vasimuddin *et al.*, 2019). The contigs were further scaffolded using the provided Hi-C data ( Rao *et al.*, 2014) in YaHS ( Zhou *et al.*, 2023) using the --break option for handling potential misassemblies. The scaffolded assemblies were evaluated using Gfastats ( Formenti *et al.*, 2022), BUSCO ( Manni *et al.*, 2021) and MERQURY.FK ( Rhie *et al.*, 2020). The mitochondrial genome was assembled using MitoHiFi ( Uliano-Silva *et al.*, 2023), which runs MitoFinder ( Allio *et al.*, 2020) and uses these annotations to select the final mitochondrial contig and to ensure the general quality of the sequence.

The assembly was checked for contamination and corrected using the gEVAL system ( Chow *et al.*, 2016) as described previously ( Howe *et al.*, 2021). Manual curation was performed using gEVAL, HiGlass ( Kerpedjiev *et al.*, 2018) and PretextView ( Harry, 2022).

### Evaluation of final assembly

A Hi-C map for the final assembly was produced using bwa-mem2 ( Vasimuddin *et al.*, 2019) in the Cooler file format ( Abdennur & Mirny, 2020). To assess the assembly metrics, the *k*-mer completeness and QV consensus quality values were calculated in Merqury ( Rhie *et al.*, 2020). This work was done using Nextflow ( Di Tommaso *et al.*, 2017) DSL2 pipelines “sanger-tol/readmapping” ( Surana *et al.*, 2023a) and “sanger-tol/genomenote” ( Surana *et al.*, 2023b). The genome was analysed within the BlobToolKit environment ( Challis *et al.*, 2020) and BUSCO scores ( Manni *et al.*, 2021; Simão *et al.*, 2015) were calculated.

Table 4 contains a list of relevant software tool versions and sources.

**Table 4.** Software tools: versions and sources.

Software tool	Version	Source
BlobToolKit	4.2.1	https://github.com/blobtoolkit/blobtoolkit
BUSCO	5.3.2	https://gitlab.com/ezlab/busco
bwa-mem2	2.2.1	https://github.com/bwa-mem2/bwa-mem2
Hifiasm	0.16.1-r375	https://github.com/chhylp123/hifiasm
HiGlass	1.11.6	https://github.com/higlass/higlass
Merqury	MerquryFK	https://github.com/thegenemyers/MERQURY.FK
MitoHiFi	2	https://github.com/marcelauliano/MitoHiFi
PretextView	0.2	https://github.com/sanger-tol/PretextView
purge_dups	1.2.3	https://github.com/dfguan/purge_dups
sanger-tol/ascc	-	https://github.com/sanger-tol/ascc
sanger-tol/ genomenote	v1.0	https://github.com/sanger-tol/genomenote
sanger-tol/ readmapping	1.1.0	https://github.com/sanger-tol/readmapping/tree/1.1.0
YaHS	yahs-1.1.91eebc2	https://github.com/c-zhou/yahs

### Genome annotation

The Ensembl Genebuild annotation system ( Aken *et al.*, 2016) was used to generate annotation for the *Andrena trimmerana* assembly (GCA_951215215.1) in Ensembl Rapid Release at the EBI. Annotation was created primarily through alignment of transcriptomic data to the genome, with gap filling via protein-to-genome alignments of a select set of proteins from UniProt ( UniProt Consortium, 2019).

### Wellcome Sanger Institute – Legal and Governance

The materials that have contributed to this genome note have been supplied by a Darwin Tree of Life Partner. The submission of materials by a Darwin Tree of Life Partner is subject to the **‘Darwin Tree of Life Project Sampling Code of Practice’**, which can be found in full on the Darwin Tree of Life website here. By agreeing with and signing up to the Sampling Code of Practice, the Darwin Tree of Life Partner agrees they will meet the legal and ethical requirements and standards set out within this document in respect of all samples acquired for, and supplied to, the Darwin Tree of Life Project.

Further, the Wellcome Sanger Institute employs a process whereby due diligence is carried out proportionate to the nature of the materials themselves, and the circumstances under which they have been/are to be collected and provided for use. The purpose of this is to address and mitigate any potential legal and/or ethical implications of receipt and use of the materials as part of the research project, and to ensure that in doing so we align with best practice wherever possible. The overarching areas of consideration are:

•    Ethical review of provenance and sourcing of the material

•    Legality of collection, transfer and use (national and international)

Each transfer of samples is further undertaken according to a Research Collaboration Agreement or Material Transfer Agreement entered into by the Darwin Tree of Life Partner, Genome Research Limited (operating as the Wellcome Sanger Institute), and in some circumstances other Darwin Tree of Life collaborators.

## Data Availability

European Nucleotide Archive: *Andrena trimmerana* (trimmer’s mining bee). Accession number PRJEB56904; https://identifiers.org/ena.embl/PRJEB56904. The genome sequence is released openly for reuse. The *Andrena trimmerana* genome sequencing initiative is part of the Darwin Tree of Life (DToL) project. All raw sequence data and the assembly have been deposited in INSDC databases. Raw data and assembly accession identifiers are reported in Table 1.

## Author information

Members of the University of Oxford and Wytham Woods Genome Acquisition Lab are listed here: https://doi.org/10.5281/zenodo.7125292.

Members of the Darwin Tree of Life Barcoding collective are listed here: https://doi.org/10.5281/zenodo.4893703.

Members of the Wellcome Sanger Institute Tree of Life Management, Samples and Laboratory team are listed here: https://doi.org/10.5281/zenodo.10066175.

Members of Wellcome Sanger Institute Scientific Operations: Sequencing Operations are listed here: https://doi.org/10.5281/zenodo.10043364.

Members of the Wellcome Sanger Institute Tree of Life Core Informatics team are listed here: https://doi.org/10.5281/zenodo.10066637.

Members of the Tree of Life Core Informatics collective are listed here: https://doi.org/10.5281/zenodo.5013541.

Members of the Darwin Tree of Life Consortium are listed here: https://doi.org/10.5281/zenodo.4783558.
