# Supplementary material for: The genome sequence of Trimmer's Mining Bee, Andrena trimmerana (Kirby, 1802)
Source: Wellcome Open Res. 2024 Nov 22;9:691. [Version 1] doi: 10.12688/wellcomeopenres.23370.1 (PMC12596548; doi:10.12688/wellcomeopenres.23370.1)
Supplement: Peer review history

# Reviewer report: Reviewer response for version 1

**DOI:** 10.21956/wellcomeopenres.25765.r136969

**Published:** 2025-11-13

**Authors:** Josephine R Paris

**Affiliations:** 1 University of Exeter Library, Devon, UK

This is a well-written Data Note describing the genome assembly and annotation of the Trimmer's Mining Bee. The Background information provides thorough information on the biology and ecology of the species. The genome assembly lab and bioinformatics protocols are standard. Data can be found on the ENA and Ensembl Rapid Release.

 I have only a few minor comments:

 Abstract: there's a space missing: "afemale specimen" should be "a female specimen"

 Background: please include the latin names: willow, dandelion, gorse, bramble, blackthorn, alexanders, and rhododendron

 Report: please explain why only 86.18% of the assembly was assigned to 5 chromosomes. Were there difficulties in the curation? Was the data coverage too low? This will help others experiencing similar issues

Are sufficient details of methods and materials provided to allow replication by others?

Yes

Is the rationale for creating the dataset(s) clearly described?

Yes

Are the datasets clearly presented in a useable and accessible format?

Yes

Are the protocols appropriate and is the work technically sound?

Partly

Reviewer Expertise:

population genomics of non-model organisms, including genome assembly and annotation

I confirm that I have read this submission and believe that I have an appropriate level of expertise to confirm that it is of an acceptable scientific standard, however I have significant reservations, as outlined above.

# Reviewer report: Reviewer response for version 1

**DOI:** 10.21956/wellcomeopenres.25765.r136974

**Published:** 2025-11-08

**Authors:** Liang Lu

**Affiliations:** 1 Hebei Normal University, Shijiazhuang, Hebei, China

The manuscript presents a high-quality HiFi genome assembly of *Andrena trimmerana* (Kirby, 1802), complemented by Hi-C based chromosomal scaffolding. Structurally, it follows the established format of previous Wellcome Open Research genome reports, offering comprehensive background on taxonomy, biology, and specimen collection. The inclusion of clear diagnostic images enhances confidence in species identification. The methodologies for sample preparation, DNA extraction, sequencing, assembly, and annotation are consistent with the journal’s prior publications and appear technically sound.

 However, I would encourage the authors to consider consolidating future genome reports—perhaps by publishing multiple assemblies or a thematic package within a single manuscript or a limited series. Much of the content in these reports is standardized and could be efficiently presented in a more aggregated format.

 One tiny omission is the lack of detail regarding tissue sampling. Table 1 indicates that sequencing was performed on the three tagmata using different technologies, but it remains unclear whether entire tagmata were used or if specific tissues (e.g., muscle) were selectively processed. Furthermore, the manuscript does not address how intestinal contents or potential intracellular parasites and symbionts were handled. Clarification is needed on whether post-assembly decontamination procedures were sufficient to remove non-host sequences originating from these sources.

Are sufficient details of methods and materials provided to allow replication by others?

Yes

Is the rationale for creating the dataset(s) clearly described?

Yes

Are the datasets clearly presented in a useable and accessible format?

Yes

Are the protocols appropriate and is the work technically sound?

Yes

Reviewer Expertise:

phylogenomics, entomology

I confirm that I have read this submission and believe that I have an appropriate level of expertise to confirm that it is of an acceptable scientific standard.

# Reviewer report: Reviewer response for version 1

**DOI:** 10.21956/wellcomeopenres.25765.r114036

**Published:** 2024-12-30

**Authors:** Rodolpho S. T. Menezes

**Affiliations:** 1 State University of Santa Cruz, Ilhéus, Brazil

The data note by Baker et al. presents a genome assembly for Trimmer’s Mining Bee, *Andrena trimmerana* (Kirby, 1802), with a total sequence length of 399.10 megabases. This genome provides valuable insights for comparative genomics, as well as evolutionary and conservation studies. The methodology is comprehensive and well-documented, while the figures are clearly constructed, contributing to a well-written data note.

 However, I recommend that the authors include more detailed information about the mitogenome. Additionally, a brief comparison of key descriptive statistics with the recent genome assembly of the Small Scabious Mining Bee, *Andrena marginata*, would enhance the note by providing context and broader relevance.

Are sufficient details of methods and materials provided to allow replication by others?

Yes

Is the rationale for creating the dataset(s) clearly described?

Yes

Are the datasets clearly presented in a useable and accessible format?

Yes

Are the protocols appropriate and is the work technically sound?

Yes

Reviewer Expertise:

Phylogenomics, Cytogenetics, Phylogeography.

I confirm that I have read this submission and believe that I have an appropriate level of expertise to confirm that it is of an acceptable scientific standard.
